# scRNA sequencing revealed HIV-associated inflammation-mediated lung epithelial dysregulation and fibroblast remodeling

**DOI:** 10.3389/fimmu.2026.1789140

**Published:** 2026-04-16

**Authors:** Khursheed Ul Islam, Gagandeep Kaur, Sadiya Bi Shaikh, Kingshuk Panda, Srinivasan Chinnapaiyan, Hoshang Jehangir Unwalla, Irfan Rahman

**Affiliations:** 1 Department of Environmental Medicine, University of Rochester Medical Center, Rochester, NY, United States; 2 Department of Cellular and Molecular Medicine, Herbert Wertheim College of Medicine, Florida International University, Miami, FL, United States

**Keywords:** cell heterogeneity, chronic inflammation, epithelial to mesenchymal transition, HIV, lung injury, single cell RNA sequencing

## Abstract

**Background:**

HIV infection is risk factor for a wide spectrum of pulmonary diseases, with incidence rates significantly higher in people living with HIV (PLWH). Despite antiretroviral therapy, persistent immune activation and recurrent injury continue to compromise lung integrity in this population. However, the mechanism by which HIV disrupts alveolar homeostasis and affects epithelial, immune, and stromal compartments in the lungs remain undefined.

**Objective:**

To characterize the cellular and molecular landscape of HIV infection within the lungs, the focus of the study was to assess epithelial remodeling and HIV-driven alterations in lung cellular composition and transcriptional programs.

**Methods:**

Single cell RNA sequencing (scRNA-seq) was performed on human lung tissues obtained from HIV infected and uninfected individuals, including both non-smokers and smokers. A total of 54,230 cells across all experimental groups were analyzed using integration and Uniform Manifold Approximation and Projection (UMAP) clustering to identify transcriptionally distinct cell populations and HIV-associated changes.

**Results:**

HIV infection profoundly altered lung cellular composition marked by expansion of CD4^+^, CD8^+^ T-cells, B cells, and non-classical monocytes, alongside reduced fibroblast abundance. Alveolar Type I and Type II cells displayed robust HIV-associated transcriptional reprogramming. Fibroblast and smooth muscle cells showed enhanced proinflammatory and stress responsiveness affecting extracellular matrix and contractile programs. EMT marker analysis revealed cell type specific shifts with: (a) AT1 cells exhibiting reduced *CDH1* and increased vimentin gene expression, (b) AT2 cells adopting a hybrid epithelial mesenchymal phenotype, and (c) myofibroblast cluster displaying amplified mesenchymal activation.

**Conclusion:**

These findings revealed HIV as a potential driver of epithelial dysregulation and airway remodeling in the human lungs. These observations provide a framework for future studies aimed at determining whether modulation of these pathways may have therapeutic relevance, particularly in the context of lung pathology in PLWH.

## Introduction

Infection caused by Human immunodeficiency virus (HIV) remains a major global health concern. Even with the success of combination antiretroviral therapy (cART), people living with HIV (PLWH) experience a range of chronic comorbidities, including progressive lung diseases (1). The lungs represent an important site of HIV-related injury characterized by persistent inflammation and epithelial disorganization (2). The airway and alveolar epithelium form a critical barrier that protects against inhaled pathogens and environmental stressors, while alveolar type 1 (AT1) and type 2 (AT2) cells maintain gas exchange and repair following injury (3). Disruption of this epithelial barrier can have profound effects on pulmonary homeostasis, integrity, and permeability during HIV infection (4, 5). Increasing evidence suggests that HIV can infect lung epithelial cells, albeit at a lower efficiency than the classical immune targets such as CD4^+^ T cells and macrophages (4).

HIV infection is also associated with persistent pulmonary immune activation characterized by chronic inflammation within the alveolar environment. This inflammatory state is driven in part by increased numbers of activated alveolar macrophages and infiltrating CD8^+^ T cells, both of which accumulate in the lungs (5). Chronic activation of alveolar macrophages and CD8^+^ T cells leads to the release of proinflammatory cytokines, chemokines, and oxidative mediators that can compromise epithelial barrier integrity, disrupt tight junction architecture, and induce cellular stress. Moreover, HIV proteins, such as tat and gp120 induce epithelial mesenchymal transition (EMT) in oral and genital epithelial cells through activation of TGF-β and MAPK signaling (6). HIV as well as its associated proteins like HIV tat and nef (negative regulatory factor) proteins are known to alter epithelial integrity even in non-positive bystander cells (7, 8). Cigarette smoking and HIV both promote chronic inflammation and immune dysfunction independently within the lung microenvironment (9). However, how these factors interact at the molecular level to shape the lung pathology remains elusive.

To address this knowledge gap, we examined lung tissues derived from HIV negative and HIV positive individuals with and without a history of smoking to identify key pathways altered by HIV and cigarette smoking. We hypothesize that HIV infection promotes a state of chronic inflammation and immune dysregulation within the lungs, which may contribute to progressive tissue injury and structural alterations over time. To better understand how HIV infection and cigarette smoking affect the cellular and molecular landscape of the human lungs, we employed single cell RNA sequencing (scRNA seq) to profile lung tissues from HIV positive and non-HIV individuals with varying smoking histories. scRNA seq provides an unprecedented opportunity to dissect the cellular heterogeneity and transcriptional dynamics of complex tissues at single-cell resolution (10). This high-throughput approach allowed us to examine the effects of HIV across individual epithelial, immune and stromal cell populations, providing insight into cell type specific transcriptional alterations that may underline HIV-associated lung remodeling. To support key pathway level observations derived from the scRNA seq dataset, we performed complementary validation assays including western blotting and multiplexed ELISA. Together, this integrated strategy establishes framework for future work to study the cellular and molecular consequences of HIV infection in the lungs and lays the foundation for future mechanistic studies aimed at understanding the drivers of chronic lung injury in PLWH.

## Materials and methods

### Ethics statement

All the experiments in this study were performed according to the biosafety guidelines approved by the University of Rochester institutional biosafety committee.

### Ethics approval

The human patients and the patients’ data included in the study were procured from National Disease and Research Interchange (NDRI), as human subject recruitment was not directly involved with this work. No consent was provided because these lung tissues were anonymous, archival, and de-identified discarded tissues. The procurement of human lung tissue samples as de-identified samples was approved by the Materials Transfer Agreement (Institutional Review Board, IRB) with exemption on October 5, 2021 via our RSRB office Study (ID: STUDY00006571; is not research involving human subjects as defined by DHHS and FDA regulations), along with laboratory protocols by the Institutional Biosafety Committee (IBC) at the University of Rochester Medical Center, Rochester, NY.

All other procedures/protocols were carried out per the guidelines and regulations specified by the University of Rochester, Rochester, NY.

### Tissue collection and preparation

We employed lung tissue collected from healthy (Non-smokers and Smokers) and diseased (HIV with and without smoking history) human subjects as samples (n = 3-4/group). The HIV status of these lungs were confirmed by immunostaining tissues with HIV-Tat as shown previously (11). A representative image showing HIV Tat staining in HIV-infected tissues is shown in the supplementary data (**Supplementary Figure 1**). Total of 15 lung tissue samples were chosen for this study. The samples procured were validated for their disease categories based on their clinical status as provided by NDRI. Frozen lung tissue samples from age-matched (43–53 years) HIV infected and healthy subjects were grouped according to their smoking status at the time of collection. A detailed characteristic of the lung tissue samples used for this study is given in **Table 1**. Other important information available for the donor samples is also presented in the supplementary results showing comorbidities, viral load, CD4^+^ counts and smoking intensities (**Supplementary Table 1**).

**Table 1:** Clinical characteristics of the samples used for the scRNA seq analyses.

Characteristics	HIV-		HIV+	
	Non-smokers	Smokers	Non-smokers	Smokers
n	4	4	4	3
Male Sex (%)	50	50	40	33.3
Age (Mean±SD)	50.75 ± 6.24	48.25 + 8.02	48.5 ± 3.51	43.7 ± 10.97
Demographics				
White(%)	100	100	50	66.7
Black(%)	0	0	25	33.3
Hispanic(%)	0	0	25	0

### Preparation of single cell suspension

Approximately 40–50 mg of frozen lung tissue samples were employed for preparation of single cell suspension to perform single-cell RNA sequencing (scRNA seq). The tissue processing and scRNA seq were outsourced and performed by Singulomics Corporation at Albert Einstein College of Medicine, Bronx, New York, USA. In brief, flash-frozen tissues were first chemically fixed to preserve cellular integrity and RNA content. The tissues were minced into small pieces using a sterile blade on a glass surface and transferred to the fixation buffer at a ratio of 1 mL per 25 mg of tissue. The samples were incubated at 4 °C for 24 hours without agitation. Following fixation, tissues were washed with PBS and subsequently quenched with the quenching buffer provided in the kit to neutralize the fixative. The fixed tissues were enzymatically digested using a pre-warmed dissociation solution containing the Liberase enzyme and mechanically dissociated into single-cell suspensions using the gentleMACS Octo Dissociator (Miltenyi Biotech), ensuring optimal cell viability and minimal aggregation. The single-cell suspension was incubated with a pool of barcoded probe pairs designed to target RNA molecules within a controlled hybridization reaction. After an overnight incubation, unbound probes were removed through a series of wash steps, and the labeled cells were pooled before proceeding to microfluidic partitioning.

### Single cell RNA sequencing

#### Library preparation

10x Genomics Chromium Fixed RNA profiling Reagent Kit (10x Genomics, Pleasanton, CA) was employed for library preparations in single cell suspension of our samples. 10,000 cells per sample were captured using 10X Chromium single cell platform and sequenced using NoVaSeq 6000 (Illumina, San Diego, CA) maintaining a mean sequence depth of 30,000 reads per cell providing comprehensive transcriptome coverage. The read alignment was performed to GRCh38-2024-A human reference genome. Intronic reads were included in the analysis to capture both the nascent and mature RNA transcripts, thereby enhancing the resolution of gene expression dynamics.

### Data processing and analysis

To analyze the single cell RNA seq data we used Seurat v5.1.0 analyses pipeline (12). The analyses dataset was created by excluding the low-quality cells (nFeature RNA < 200 & percent.mt > 1e+05) and potential doublets. All the datasets were integrated using RunHarmony algorithm followed by data normalization of integrated data using standard Seurat pipeline (12). Moreover, Identification of the variable genes for dimensionality reduction was done using “FindVariableGenes” gene function using PCA function. The cell type annotation through Azimuth pipeline using the Human-Lung v2 (HLCA) reference dataset (13) was performed. Cell clusters displaying cell counts less than 150 cells were excluded from further analysis as these may be representative of arbitrary changes and would not yield statistically significant results. **Supplementary Figure 2** shows a flowchart with the basic steps used for data preprocessing, Normalization and post-clustering analyses with the threshold and statistical criteria employed for this study.

### Differential expression and gene enrichment analyses

Identification of differentially expressed genes (DEG) was done using DESeq2 (1.42.1) package by performing pseudobulk analyses. This program uses negative binomial generalized linear model (GLM) to predict counts matrix for each gene and for the calculation of log2 fold changes. Following differential expression analysis, genes with an adjusted p-value (Padj) <0.05 were considered statistically significant. The significant differentially expressed genes (DEGs) were then sorted in an ascending order based on their expression values. A heatmap was subsequently generated to visualize the expression patterns of these DEGs, using their respective fold change values to illustrate the relative upregulation and downregulation across samples.

Functional enrichment analysis of differentially expressed genes (DEGs) was conducted using the ClusterProfiler R package (v4.10.1). For each cell type, the identified DEGs were analyzed for enrichment across all Gene Ontology (GO) categories, including Biological process (BP), Molecular Function (MF), and Cellular Component (CC). Ensemble (14) gene symbols were used as input identifiers. The significant up and downregulated genes were identified as per the condition log2FoldChange > 0.5 and log2FoldChange < -0.5. Enrichment significance was determined using the default settings for enrichGO with a p-value threshold of p < 0.05 and a false discovery rate (FDR, Benjamini–Hochberg) threshold of q < 0.2.

### Western blotting

Homogenates from lung tissues (Healthy and patients) were subjected to protein isolation using RIPA lysis buffer (Cat# 20-188, Millipore Sigma). Briefly, 50-100mg of frozen lung tissue was minced into small pieces and homogenized in 400 µl of ice-cold RIPA lysis buffer using an electric homogenizer. The microcentrifuge tube was kept on ice throughout the homogenization process to maintain protein stability. Following homogenization, tissue homogenates were incubated for 30 min over ice and then centrifuged at 13000 RPM for 30 minutes at 4 °C. The supernatant was transferred to a fresh 1.5mL Eppendorf tube and subjected to protein estimation using BCA protein Assay Kit (Cat# 23225, Thermo scientific). 20 μg of proteins per sample were loaded and resolved using 10-12% Sodium dodecyl sulphate-polyacrylamide gel electrophoresis (SDS-PAGE). Proteins separated through SDS-PAGE were then transferred to PVDF membrane (Cat# 162-0177, Bio Rad) using wet transfer method. To avoid non-specific binding to the antibodies, the blots were blocked in 5% blocking buffer solution for 1 hour at room temperature with constant agitation. The membranes were then probed with a specific primary Ab: (1:1000 dilution in 5% BSA in PBS containing 0.1% Tween 20) of Cadherin-1 (Cat# ab1416, Abcam) Vimentin (Cat# ab92547, Abcam), Fibronectin (Cat# ab2413: Abcam), and β-actin (Cat# ab20272: Abcam) at 4 °C overnight. After three washing steps (10 min each), the levels of protein were detected by probing with appropriate secondary anti-rabbit or anti-mouse antibody (1:10,000 dilution in 5% BSA) linked to HRP for 1 h, and bound complexes were detected using the ECL method (SuperSignal west Femto, ThermoFisher scientific). Equivalent loading of the gel was determined by quantitation of protein, as well as by re-probing the membranes for β-actin. The ImageJ densitometry software (Version 1.41; National Institutes of Health, Bethesda, MD) was used for gel and quantitative densitometric analysis.

### Luminex multiplex cytokine assay

Lung tissue samples from control and HIV positive patients were homogenized in ice cold lysis buffer using a tissue homogenizer as described earlier (13, 14). The homogenates were centrifuged at 12,000 × g for 15 minutes at 4 °C to remove cellular debris, and the supernatants were collected. Total protein concentration was determined using bicinchoninic acid (BCA) assay (Pierce BCA Protein Assay Kit Cat# 23225, ThermoScientific), and equal amount of protein were used for cytokine analysis.

Cytokine concentration was quantified using human cytokine magnetic bead panel (Cat# M500KCAF0Y) as described previously, following the manufacturer’s instructions (15). Briefly, supernatants from tissue homogenates were quantified by BCA method and diluted 1:4 in the provided diluent buffer. Equal protein concentrations (40 μg per well) were loaded, and samples were incubated with fluorescent labelled magnetic beads conjugated to cytokine specific capture antibodies. After washing to remove the unbound proteins, biotinylated detection antibodies and streptavidin- phycoerythrin were added to generate fluorescent signals proportional to the amount of cytokine bound. Samples were acquired on a Luminex MAGPIX system (XMAP Technology) and data were analyzed. Cytokine concentration was calculated from a standard curve generated using recombinant cytokine standard supplied with the kit. All samples were run in duplicates, and values were normalized to total protein concentration.

### Statistical analyses

Statistical analyses were conducted using GraphPad Prism 10.5.0. Results are reported as mean ± SEM. Pairwise comparisons were analyzed using unpaired *t*-tests, and one-way ANOVA with Tukey’s *post hoc* test was used for multi-group analyses.

## Results

### Single-cell RNA Sequencing reveals distinct cellular landscape in HIV-infected lungs

**Figure 1:**
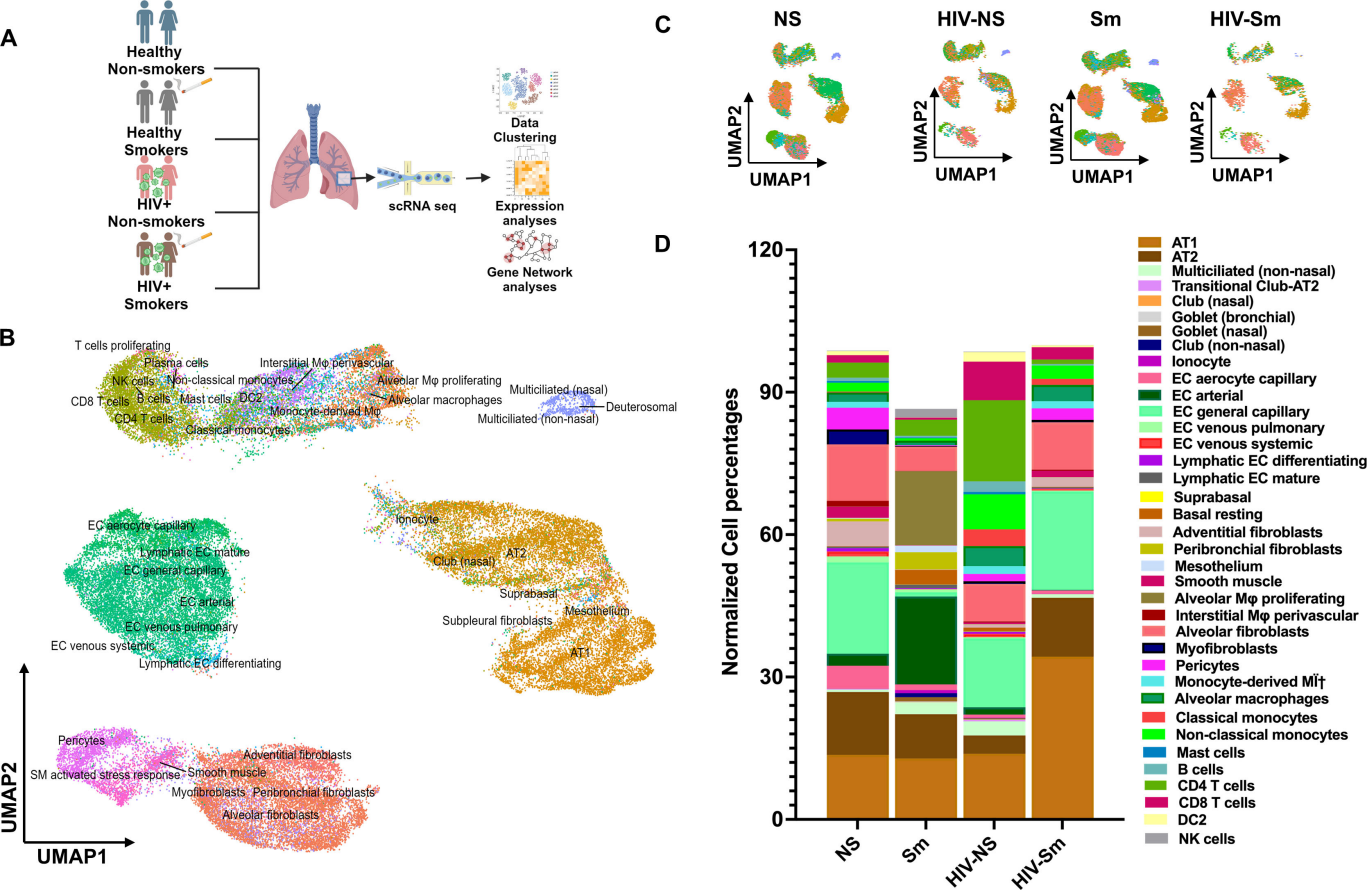
Dimensionality reduction identifies 29 unique cluster and increase in AT1 cells in HIV patients with a smoking history. Frozen lung tissue samples from HIV donors with and without a smoking history and non-diseased smokers and non-smokers were used to perform single cell RNA sequencing (scRNA seq). **(A)** Schematics showing the study design used to identify differentially expressed genes (DEGs) and gene enrichment analyses. **(B)** Uniform Manifold Approximation and Projection (UMAP) clustered 54230 cells into 29 distinct clusters using Azimuth annotations. **(C)** UMAP plot representation of all the cell types within diseased (HIV+) and non-diseased smokers (Sm) and non-smokers (NS). **(D)** The cell frequency plot showing the cell type distributions within HIV-Sm, HIV-NS, Sm and NS groups.

HIV positive individuals remain at high risk of worsened lung functions and for the development of chronic lung diseases, such as COPD, asthma, pneumonia and lung cancer. To investigate the effect of HIV infection on lung cellular composition, we performed single-cell RNA sequencing (scRNA-seq) on lung tissues obtained from: healthy non-smokers (NS) and HIV-positive individuals without a history of smoking (HIV-NS). In addition, considering that smoking can have a unique heterogeneous effect on the diseased lungs, thereby leading to accelerated progression of chronic lung ailments (COPD and fibrosis) in HIV patients, we also included samples from HIV-positive and HIV-negative individuals with a history of chronic smoking (>20 pack years) in our study cohort. Transcriptional profiles from these samples were integrated and analyzed to assess cellular heterogeneity as described earlier (10). Of note, all the samples underwent a stringent quality check as described in the Methods section and only the nCounts and nFeatures meeting the pre-filter quality checks were included in the study. **Supplementary Figure 3** shows the violin plot depicting the distribution of QC metric parameters (total reads/nCounts, number of features/nFeatures, and mitochondrial percentage) for each sample before and after filtration to denote that good quality samples were included for integration in this study. Our comprehensive scRNA-seq analysis pipeline, outlined in **Figure 1A** depicts the schematic representation of study design used to identify differentially expressed genes and pathways. The scRNA-seq dataset from all the experimental groups was integrated and clustered based on the variable features. Dimensionality reduction and clustering of 54,230 cells across all experimental groups identified 29 distinct clusters, which were annotated using the Azimuth reference-based mapping pipeline (**Figure 1B**). **Supplementary Table 2** provides a detailed account of the variable features of each cluster upon integration.

Upon plotting sample-specific UMAPs for our integrated data set, we noted very low cell counts for HIV-Sm group (**Figure 1C**). This was due to low sample quality and RNA integrity number (RIN) scores for the RNA isolated from this group. This could happen due to the long storage duration of these samples (16). Owing to the low RIN score for HIV-smoker, both smoker and HIV-smoker groups were excluded from further analyses in this study. Relative abundance of different cell types within each group was visualized by generating a bar plot representing the proportional distribution of annotated cell types. As expected, we observed a notable increase in the cell frequencies CD4 and CD8 T cells in HIV infected (CD4: 17.08% & CD8: 8.14%) individuals as compared to non-smoking, healthy controls (CD4: 3.16% & CD8: 1.57%). Since the main focus of this study was to understand the effects of HIV infection on human lung tissue, we focused primarily on the changes in the proportions of alveolar epithelial cells and mesothelium in the lungs. Interestingly we observed approximately 3-fold decrease in AT2 and 2-fold decrease in alveolar fibroblast frequencies in HIV-NS group as compared to NS controls (**Figure 1D**). A loss of these two cell types may have serious implications on the epithelial-mesenchymal repair mechanisms in HIV lungs (17).

### Altered alveolar epithelial composition in HIV positive donor lungs

The gas-exchange units of human lungs are lined by alveolar epithelium which comprises of 1) AT1 cells, covering most of the alveolar surface and form a thin barrier optimized for gas diffusion, and 2) AT2 cells, which produce surfactants and serve as progenitors for epithelial repair (18). To examine changes in epithelial cell composition, we compared epithelial cell frequencies across all the groups plotted in the frequency plot. Distinct distribution of alveolar epithelial cell population was observed among the groups as discussed previously (**Figure 1D**). Distinct differences in alveolar epithelial populations were observed. Notably, HIV-positive samples demonstrated an increased proportion of cells exhibiting AT1 characteristics accompanied by relative reduction in AT2 cell frequency compared to controls. Additionally, an increased proportion of multiciliate epithelial cells was detected in HIV-positive lungs. Together these frequency shifts highlight group specific differences in epithelial cell composition associated with HIV infection.

### HIV-associated expansion of monocytes and T-cells in lung microenvironment

To further characterize changes in the pulmonary immune and stromal compartments, we compared immune and mesenchymal cell frequencies across the four experimental groups. Our results revealed a notable difference in immune cell composition (**Figure 1D**). HIV-positive lung tissues showed an increased frequency of non-classical monocytes, a subset associated with vascular surveillance and cytokine production. Expansion of both CD4^+^ and CD8^+^ T cells was also observed in HIV-positive samples compared to healthy controls, consistent with previous reports (2, 19). In parallel, HIV positive lungs exhibited a reduction in alveolar fibroblasts and myofibroblasts, two mesenchymal cell types involved in extracellular matrix organization and epithelial support (19). These observations indicate group-specific alterations in immune and stromal cell composition associated with HIV infection.

### HIV infection significantly alters the transcriptional regulation of lung epithelium and mesothelium

**Figure 2:**
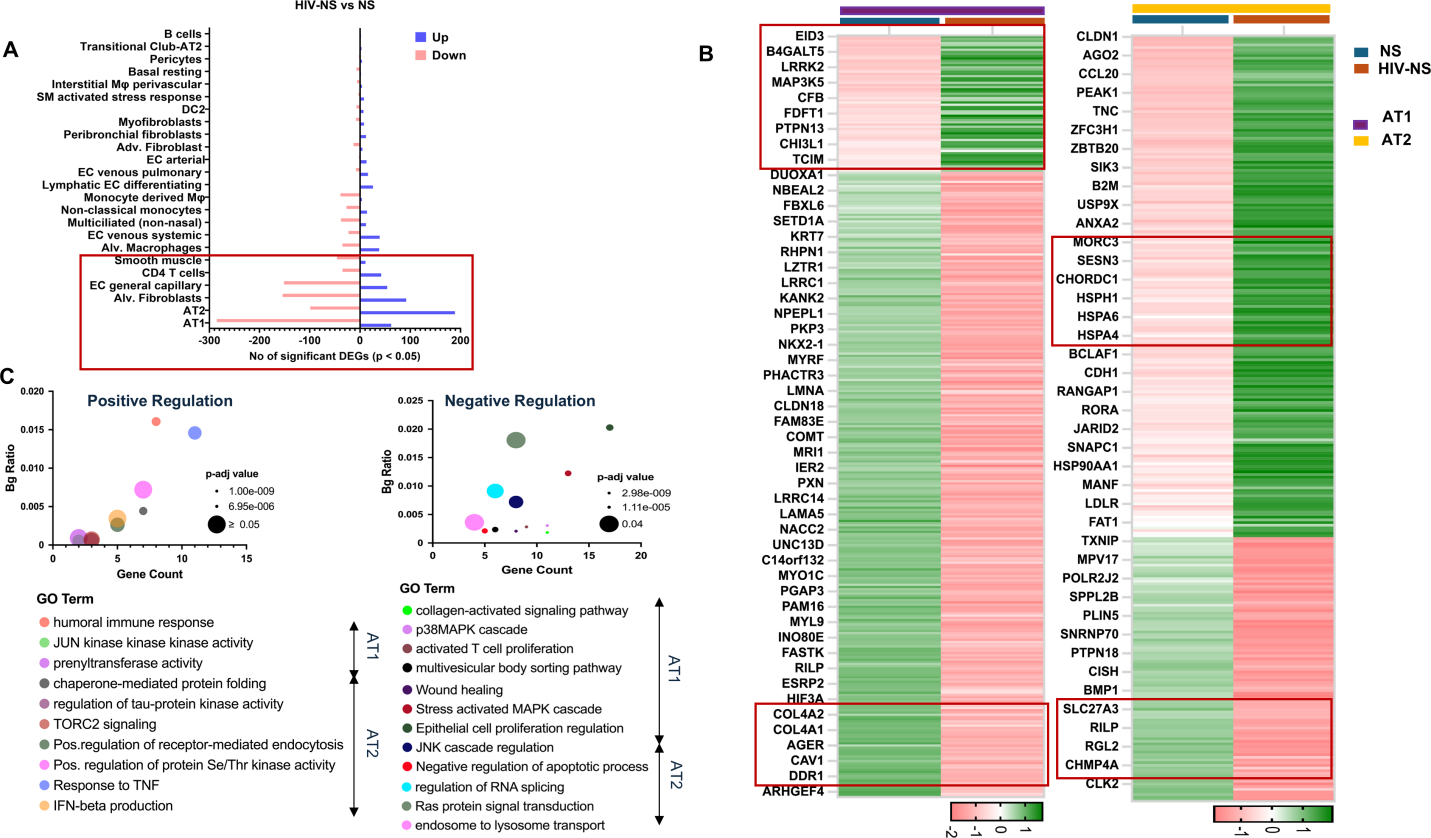
HIV infection results in dysregulation of inflammatory and apoptotic pathways. **(A)** DESeq2 analyses to identify differential gene expression in HIV patients (HIV-NS) with no smoking history compared to healthy controls identified most up (blue)-and down (red)-regulated genes in alveolar type 1(AT1), alveolar type II (AT2), alveolar fibroblast (Alv. Fibro.) and smooth muscle (SM) clusters. **(B)** Heatmap showing significant (p<0.05) DEGs between healthy controls and HIV infected samples in AT1 and AT2 cells. **(C)** Bubble plot showing the gene enrichment results of significant (p<0.05) up/ downregulated DEGs in AT1 and AT2 clusters in HIV-NS donors compared to non-smoking healthy controls.

Next, we performed differential expression analysis using DESeq2 on individual cell clusters to assess transcriptional differences between healthy non-smokers and PLWH. Although we observed a marked increase in the frequency of CD4^+^ T cells in the HIV non-smoker (HIV-NS) group, this population was not among the most transcriptionally altered clusters in the lung. In contrast, substantial transcriptional changes were detected in the AT1 and AT2 epithelial cell populations of HIV-infected individuals, with 347 (62 up- and 285 downregulated) and 288 (189 up- and 99 downregulated) significantly differentially expressed genes (p < 0.05), compared to healthy non-smoker controls. Alveolar fibroblasts, EC general capillary and smooth muscle cell clusters showed 246, 205 and 57 significant DEGs in HIV-NS group as compared to NS (**Figure 2A**). These findings indicate HIV infection broadly affects the transcriptomic landscape of both epithelial and stromal compartments in the lung.

HIV infection imposes a persistent stress burden on the alveolar epithelium, triggering both cellular adaptation and immune activation mechanisms (20). Viral proteins disrupt epithelial homeostasis, leading to oxidative stress, mitochondrial dysfunction and altered barrier integrity which further drives lung injury (21). The mechanism by which HIV infection alters alveolar epithelial homeostasis and predisposes the lung to chronic injury remains poorly understood. To this end we hypothesized that HIV imposes a persistent stress burden on alveolar epithelial cells, leading to the activation of stress responsive and immune regulatory pathways. Our single-cell RNA sequencing data confirmed this speculation by showing drastic changes in the transcriptional signatures in AT1 and AT2 cell clusters from HIV-positive lungs relative to healthy controls. Upon closer observation, we found a significant (p<0.05) upregulation of genes associated with; 1) barrier remodeling & epithelial plasticity [*CLDN1* (log2fc=3.5), *EPS8* (log2fc=2.6)], 2) inflammatory/immune activation [*CD274/PD-L1*(log2fc=4.6), *REL*(log2fc=2.7)], and 3) pro-Fibrotic/Repair mechanisms [*CHI3L1* (log2fc=6.0), *P4HA1* (log2fc=3.2)] in AT1 cell clusters from HIV-NS group as compared to NS, suggesting enhanced immune checkpoint engagement and NF-kB associated signaling within the alveolar epithelium. Furthermore, pro-fibrotic and tissue repair associated genes like *CHI3L1* and *P4HA1* upregulation indicate activation of extracellular matrix remodeling and fibrogenic pathways. In addition, genes associated with; 1. epithelial differentiation [*NKX2-1* (log2fc= -2.6), *AGER* (log2fc= -3.1), *CLDN18* (log2fc= -4.4)], 2. barrier integrity & ECM maintenance [*COL4A1* (log2fc = -2.8), *COL4A5* (log2fc= -4.0), *TIMP3*(log2fc= -2.7)], 3. stress response signaling [*FOS* (log2fc= -4.5), *FOSB* (log2fc= -5.7) and 4. Wnt Signaling [*WNT3A* (log2fc =-3.2), *SMAD*6 (log2fc= -3.1)] were found to be significantly downregulated in the AT1 cell cluster from PLWH (HIV-NS) as compared to healthy (NS) controls (**Figure 2B**) suggesting compromised maintenance of alveolar epithelial specialization and a potential disruption of barrier integrity. Similarly, suppression of stress response transcription factors and components of Wnt signaling further supports altered epithelial signaling dynamics in HIV infected lungs.

These changes are indicative of a substantial shift in the repair and stress responsiveness of the AT1 cells in disease state which may have implications leading to chronic epithelial injury, dedifferentiation or maladaptive remodeling, and early fibrotic or emphysematous susceptibility. GO analyses of significantly (p<0.05) up- and downregulated genes reported enrichment of terms like ‘humoral immune response’ (p_adj_= 0.0078), ‘JUN kinase activity’ (p_adj_= 0.021), and ‘collagen activated signaling’ (p_adj_= 2.98e-09)in AT1 cluster as shown in **Figure 2C**. A detailed account of the significantly altered DEGs and the associated GO terms is provided in **Supplementary Tables 3–10** that shows DEGs corresponding to AT1, AT2, Alveolar Fibroblasts and smooth muscle cells.

DESeq2 analyses of AT2 cell cluster identified significant (p<0.05) (a) upregulation of genes associated with inflammatory activation [*TNIP1* (log2fc= 3.46), *TNIP3* (log2fc= 8.54), *STAT4* (log2fc= 3.3)], proteastasis [*HSPD1* (log2fc= 7.64), *DNAJA4* (log2fc= 5.66)] and ECM and epithelial remodeling [*CLDN1*(log2fc= 4.39), *TNC* (log2fc= 4.85), *PLAUR* (log2fc= 3.11)] and (b) downregulation of surfactant production [*SFTPB* (log2fc= -3.03), *SLC27A3* (log2fc= -2.92), *PLA2G4F* (log2fc= -3.21)], mitochondrial and vesicle trafficking [*TXNRD2* (log2fc= -2.99), *NEURL4* (log2fc= -5.28), *RILP* (log2fc= -3.62)] and regeneration-related [*ID4* (log2fc= -3.47), *GLI4* (log2fc= -4.66), BMP1 (log2fc= -4.35)] genes in HIV-infected (HIV-NS) donor lungs as compared to controls (NS) (**Figure 2B**). Furthermore, key junctional and adhesion molecules (*CLDN1*, *CDH1*, *FAT1*) (22) and ECM remodeling mediators (*TNC, ANXA2*) (23) were enriched, suggesting an alteration in barrier stability and tissue architecture in AT2 cluster of HIV-infected lungs. Stress-adaptive proteins, including several heat shock proteins (*HSPH1, HSPA6, HSPA4, HSP90AA1*) (24) and ER stress regulators (*MANF, CHORDC1*), were also elevated, consistent with chronic injury and unfolded protein responses. Furthermore, immune-epithelial interface regulators such as CCL20 and B2M were upregulated, pointing to heightened inflammatory crosstalk (25). Epigenetic and transcriptional regulators (*JARID2, ZBTB20, USP9X*) showed increased expression, potentially diverting progenitor programs and altering differentiation trajectories (**Figure 2B**). Collectively, these signatures suggest that AT2 cells in HIV-positive lungs undergo stress-induced reprogramming, shifting their reparative role towards maladaptive remodeling and aberrant epithelial differentiation.

This was corroborated through the GO analyses of significant up- and downregulated genes from AT2 clusters. Enrichment of terms like ‘chaperone-mediated protein folding’ (p_adj_= 0.0045), ‘positive regulation of receptor-mediated endocytosis’ (p_adj_= 0.028), ‘interferon-β production’ (p_adj_= 0.048), and ‘Ras protein signal transduction’ (p_adj_= 0.04), are consistent with activation of stress-adaptive mechanisms and alteration in inflammatory signaling networks in AT2 clusters upon HIV infection (**Figure 2C**). Together, these data suggest that HIV infection reprograms AT1 and AT2 cell identities leading to impaired repair/regeneration and accelerated ECM remodeling. A detailed description of the obtained results from DESeq2 and GO analyses for AT2 cell clusters in HIV-NS vs NS group is provided in **Supplementary Tables 5–6**.

### Transcriptional and functional remodeling of alveolar fibroblasts in HIV-positive lungs

Given the transcriptional reprogramming observed in AT1 and AT2 epithelial cells (**Figure 2B**), we next investigated whether alveolar fibroblasts, a key stromal component supporting epithelial integrity, were similarly altered in HIV-positive lungs. DESeq2 analysis revealed significant upregulation of genes associated with immune activation, fibroblast remodeling, and cell death pathways (**Supplementary Figure 4A**). Notably, *SMAD5*(log2fc= 2.59) and *EDNRB* (log2fc= 2.95) were elevated, consistent with activation of TGF-β driven fibroblast remodeling and profibrotic signaling, processes that are central to fibrotic response during viral infection (26). Immune regulatory genes including *DTX4* (log2fc= 3.03), *ZNFX1* (log2fc= 2.32), *IFNGR2* (log2fc= 2.23), *CFB* (log2fc= 3.5), and *GBP3* (log2fc= 3.4) were enriched, indicating increased antiviral and pro-inflammatory responses, while *TNFRSF1B* (log2fc= 2.22) and *MLKL* (log2fc= 2.79) suggested alterations in survival and necroptotic signaling. In addition, MPP5, a regulator of epithelial polarity and cell-cell junctional organization, was upregulated, suggesting potential disruption of epithelial-mesenchymal crosstalk. In contrast, several genes were significantly downregulated in HIV-positive alveolar fibroblasts, including *ATF6B* (log2fc= -3.39), *AKT1*(log2fc= -2.4), *BAG6* (log2fc= -2.41), and *JUN* (log2fc= -3.02), which are involved in cellular stress responses, survival and transcriptional regulation. Structural and extracellular matrix related genes such as *TNXB* (log2fc= -2.63), *LAMB1*(log2fc= -2.33), *NKD2* (log2fc= -2.78), and *FIBIN* (log2fc= -4.08) were also reduced, suggesting impaired matrix organization and fibroblast-epithelial support (**Supplementary Figure 4A**). Together, these changes point towards a pro-inflammatory and pro-fibrotic phenotype in HIV-positive lungs. When considered alongside the immune and stress responsive transcriptional programs identified in AT1 and AT2 cells, these data suggest that epithelial-stromal interactions are reprogramed in HIV infection, collectively amplifying epithelial dysregulation and impairing alveolar repair.

To interpret the functional impact of these transcriptional changes, we performed pathway enrichment analysis of DEGs for alveolar fibroblasts cell cluster. The GO analyses of DEGs shows (a) positive enrichment of terms such as ‘defense response to virus (padj= 0.004)’, ‘type II interferon signaling (padj= 0.003)’, ‘tissue migration (padj= 0.010) ‘, ‘complement activation (padj= 0.0000003)’, ‘TNF response (padj= 0.043)’, and ‘serine-type peptidase (padj= 0.0007) activity’ and (b) negative enrichment of terms like ‘extracellular matrix assembly(padj= 3.16E-12)’, ‘ER-associated degradation (padj= 1.04E-07)’, ‘fatty acid metabolism (padj= 0.0006)’, and ‘TGF-β activation (padj= 6.77E-10)’ (**Supplementary Figure 4B**) in alveolar fibroblasts from HIV-infected (HIV-NS) donor lungs as compared to healthy controls (NS).

### Transcriptional remodeling of smooth muscle cells in HIV-positive lungs

In addition to fibroblast specific alterations, HIV positive lungs also demonstrated distinct transcriptional changes in smooth muscle cells (**Supplementary Figure 4A**). The DESeq2 analysis revealed the upregulation of *TAF1D* (log2fc= 2.44), *B2M* (log2fc= 4.15), *SOD2* (log2fc= 3.47), *KMT2E* (log2fc= 2.20), and *IRF1*(log2fc= 3.92) genes in smooth muscle cells of HIV-positive lungs compared to healthy controls. These genes are implicated in transcriptional regulation (*TAF1D, KMT2E*), oxidative stress response (*SOD2*), immune signaling (*B2M, IRF1*), and chromatin remodeling, suggesting that smooth muscle cells in the HIV-positive lungs adopt a more pro-inflammatory and stress-responsive phenotype. Conversely, several genes critical for extracellular matrix production (*COL1A2, EGR1, CCN1, NOTCH3*) and contractile function (*ADCY5, NR4A1, ECHDC2, ACTA2*), were significantly downregulated (p<0.05) (**Supplementary Figure 4A**), consistent with impaired smooth muscle structural integrity and tissue remodeling capacity.

GO analyses of differentially up- and downregulated genes showed enrichment of GO terms like ‘cell killing (padj= 0.0159)’, ‘T cell cytokine production (padj= 0.0057)’, ‘regulation of cellular senescence (padj= 0.0015)’, ‘cell cycle processes (padj= 0.045)’, and ‘TLR signaling (padj= 0.045)’, ‘muscle tissue development (padj= 0.001)’, ‘myofibril assembly (padj= 0.0027)’, ‘striated muscle contraction (padj= 0.0027)’, ‘integrin mediated signaling (padj= 0.020)’, and ‘smooth muscle cell proliferation (padj= 0.0189)’ in smooth muscle cluster (**Supplementary Figure 4C**).

### Immunoblotting validates the potential EMT-like phenotype and tissue remodeling in HIV infected lungs

**Figure 3:**
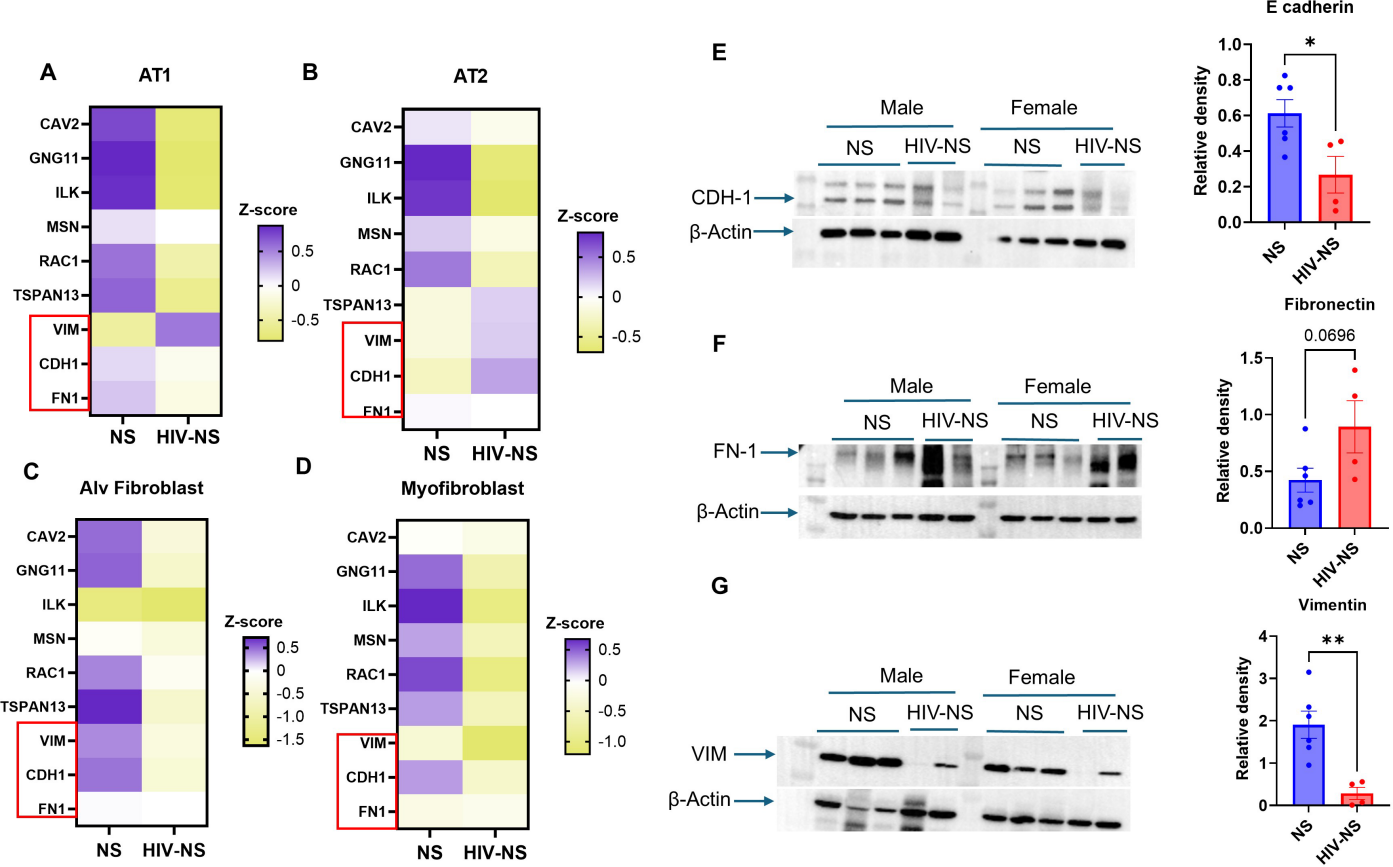
Modulation of EMT-markers during HIV infection. **(A–D)** Single cell RNA –seq analysis of EMT markers in alveolar Type1 (AT1), alveolar type 2 (AT2), alveolar fibroblasts, and myofibroblasts. In HIV-infected samples, AT1 cells exhibited increased vimentin (*VIM*) with decreased *CDH1* (CDH1) and fibronectin (*FN1*); AT2 cells showed increased *VIM* and CDH1 with no change in *FN1*; alveolar fibroblasts displayed decreased VIM and *FN1*; and myofibroblasts showed reduced *VIM* and *CDH1* with no significant change *FN1*. Bulk protein analysis of matched lung tissue lysates. **(E–G)** Representative western blots for *CDH1*, *FN1* and *VIM* with their corresponding densitometric analysis. Data represented as mean ± SEM, p-values calculated using unpaired T-test, P-values < 0.05 considered as significant.

Since we observed potential shifts in the epithelial cell identity with evidence of tissue remodeling in virus-infected donor lungs, we looked specifically into the expression of EMT-associated gene expression in our scRNA seq dataset and performed immunobotting for some select parameters in HIV-infected and healthy donor lung samples. We observed an increase in Vimentin (*VIM*) while a decrease in E-cadherin (*CDH1*) and fibronectin (*FN1*) gene transcript levels in the AT1 cell cluster from HIV-NS group as compared to NS group (**Figure 3A**) using scRNA seq data. This may suggest a shift away from epithelial identity towards a mesenchymal associated transcriptional state, an early indication of EMT in AT1 cells. Similarly, AT2 cells displayed upregulation of both *VIM* and *CDH1* expression upon HIV infection (**Figure 3B**). Conversely, we observed a marked decrease in the transcript levels of *VIM* and *CDH1* and no change in *FN1* in alveolar fibroblast and myofibroblast cluster of HIV-infected donors, further corroborating with our finding of early EMT with no signs of fibrosis in the infected tissues used for this study (**Figures 3C, D**). Full blots are shown in **Supplementary Figures 5–7**.

In line with transcriptional trends observed in scRNA seq data western blot of the whole lung tissue lysates demonstrated alteration in protein abundance associated with EMT in HIV-positive lungs compared to healthy controls. We observed significant (p value= 0.025) decrease in the CDH1 protein while an increase in fibronectin (p value=0.069) protein abundance in HIV-infected donor lungs as compared to healthy controls. Notably, the abundance of vimentin decreased in the HIV positive tissues compared to healthy controls, further corroborating with our findings from scRNA seq suggesting early EMT and tissue remodeling upon HIV infection in human lungs (27, 28).

### Cytokine profiling reveals heightened inflammatory signaling in HIV-positive lungs

**Figure 4:**
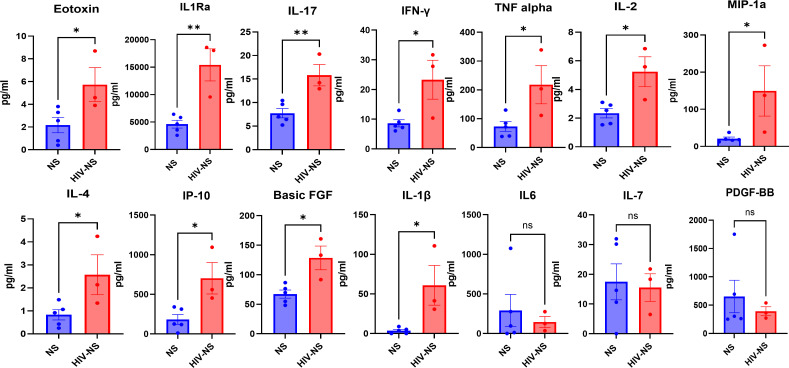
HIV associated cytokine milieu promotes epithelial-to-mesenchymal transition. Quantification of cytokine levels by Luminex assay shows significant elevated levels of IL-1β,IL-17, TNF-α, IFN-γ/IP-10, IL-4 cytokines known to drive EMT and epithelial dysfunction, alongside elevated IL-1Ra in HIV-infected lung tissues(n=3) compared to healthy controls (n=4). On the other hand, IL6, IL-7 and PDGF-BB levels are decreased in HIV-infected lung tissues as compared to healthy controls. Data represented as mean ± SEM, p-values calculated using unpaired T-test, P-values < 0.05 considered as significant.

To determine whether the transcriptional and protein-level dysregulation observed in epithelial and stromal compartments was associated with changes in the inflammatory microenvironment., we performed Luminex-based multiplex cytokine profiling on lung tissue lysates. HIV positive lungs exhibited a broad and significant increase in proinflammatory and immunomodulatory cytokine levels, including eotaxin, IL-1Ra, IL-17, IFN-γ, TNF-α, IL-2, MIP-1α, IP-10, basic FGF, and IL-1β, compared to healthy controls (**Figure 4**). Many of these cytokines are well established drivers of epithelial and fibroblast activation, with IL-1β, TNF-α, IFN-γ previously implicated in disrupting epithelial junction integrity and promoting EMT-like changes, while IL-17 and IP-10 linked to chronic immune mediated tissue remodeling (29, 30).

The upregulation of IL-1Ra and IL-4 suggests the simultaneous induction of counter regulatory pathways, reflecting a complex cytokine network in which pro-inflammatory and reparative signals coexist but may remain insufficient to restore tissue homeostasis (**Figure 4**). Elevated basic FGF further points to aberrant growth factor signaling that could contribute to stromal cell activation and fibrosis.

## Discussion

Single cell transcriptomic profiling of healthy and patient-derived lung tissues presented here provides a detailed view of how HIV infection alters the cellular composition and transcriptional state of the human lungs. These findings reveal widespread immune activation, epithelial stress, and mesenchymal reorganization, indicating a shift in lung homeostasis toward a persistently disturbed state that may promote chronic inflammation and tissue remodeling. However, given the reliance on frozen specimens and the compromised sample quality in the HIV-smoker group, findings related to this group should be interpreted as exploratory.

A striking feature of the HIV-positive lungs was the expansion in the frequencies of immune subsets, including CD4^+^, CD8^+^ T-cells and B cells. Although systematic depletion of CD4^+^ T cells is well established hallmark of HIV infection, the increased representation of these immune subsets in the lung suggests local proliferation, retention, or recruitment (29). This localized enrichment may reflect ongoing immune surveillance or viral persistence within the tissue niches. Parallelly, increased frequency of non-classical monocytes supports the notion that HIV-positive lungs maintain a heightened immune tone, potentially contributing to chronic-low grade inflammation (30).

Within the alveolar epithelium, both type-1 (AT1) and Type-2 (AT2) epithelial cells displayed transcriptional evidence of stress adaptation and immune activation. AT1 cells in HIV-positive lungs upregulated genes such as *CHI3L1, LRRK2*, *MAP3K5*, and *TCIM*, which are associated with oxidative stress responses, inflammatory signaling, and epithelial plasticity. The induction of these genes suggests that AT1 cells may acquire adaptive programs that help them persist under chronic stress but may also alter their barrier and signaling functions. AT2 cells, in contrast, exhibited increased expression of junctional proteins, ECM components, and heat shock chaperones, consistent with a state of persistent stress and altered differentiation potential. Together, these signatures suggest that HIV Infection may shift alveolar repair towards maladaptive remodeling, although this hypothesis remains to be tested in functional models.

The stromal compartments exhibited similar patterns of activation and reprogramming. Alveolar fibroblasts from HIV-positive lungs showed increased expression of immune regulatory and profibrotic genes, including *SMAD5, IFNGR2*, and *GBP3*, alongside reduced expression of the matrix and structural components such as *LAMB1* and *TNXB*. This transcriptional profile may reflect an immune-sensitized fibroblast state that contributes to the inflammatory milieu rather than maintaining structural support. Likewise smooth muscle cells exhibited upregulation of stress and immune related genes (*SOD2, B2M, IRF1*) and reduced expression of contractile genes (*ACTA2, COL1A2*), suggesting possible alteration in tissue integrity and signaling interactions with neighboring cells. These stromal responses may further amplify epithelial stress, forming a feed-forward loop between immune activation and tissue remodeling.

Evidence of partial epithelial-mesenchymal transition (EMT) programs adds another layer to this complexity. Differential expression of canonical EMT markers across AT1, AT2, and fibroblast populations suggest that HIV infection may induce a spectrum of epithelial plasticity states rather than a uniform transition. It is important to mention here that while our transcriptional (scRNA seq) and translational (immunoblotting) results may not align entirely, both point towards the same eventuality. While we see cell-specific alterations in the EMT-associated (CDH1, *VIM*, *FN1*) gene expressions through our scRNA seq data, the common thread here was the evidence of early EMT and tissue remodeling without much evidence of fibrosis in the tissue. The increased protein abundance of FN1 along with the decrease in abundances of CDH1 and VIM at whole-tissue level in HIV-infected donor lungs substantiates the same finding at translational level. While we do not assess it here, there is an element of post-transcriptional regulation of gene expressions at a cellular level, that may have a crucial role to play in governing the changes in the translation and accumulation of various proteins within the tissue.

It is further pertinent to mention that we intend to compare the changes within the lungs of HIV-infected individuals with and without a history of smoking. Owing to the bad read count for the HIV-smoker group, such variations were not explored in much detail. However, our results point towards the possibility of accelerated damage and disease progression upon external stressors, like cigarette smoke, in patients infected with HIV due to the drastic variations observed in the transcriptional homeostasis of lung epithelium and mesothelium upon infection.

Despite its key observation of EMT and tissue remodeling upon HIV infection in human lungs, our study had some limitations. At first, analyses involving the HIV-smoker subgroup were constrained by sample size and low RNA quality, reducing the robustness of the conclusions regarding smoking-HIV interaction effects. Future studies will utilize freshly collected tissue samples from this group to minimize potential compromises in sample integrity associated with prolonged cryopreservation and tar deposition due to smoking. Second, the human lung tissues used in this study were obtained as de-identified donor tissues through a third-party. Thus, a detailed clinical characterization of each donor with details including viral load, ART status, duration of infection, and pulmonary function measures, were not readily available. Consequently, the present analyses could not incorporate these potentially relevant clinical variables. The findings should therefore be interpreted as reflecting associations rather than establishing direct causal relationships. Third, the limited sample size constrained our ability to perform complex statistical tests and identify potential changes with confidence. As a result, the influence of donor heterogeneity cannot be fully excluded, and these findings should be interpreted with this limitation in mind. Future studies in larger, demographically balanced cohorts will be important for validating and extending these observations. Additionally, because tissue sampling was not consistently obtained from the same anatomical lung region, regional heterogeneity may have contributed to variability in cellular composition and transcriptional profiles.

Overall, the single-cell transcriptomic landscape presented here underscores the breadth of HIV-associated alterations in human lung, reflecting coordinated changes across diverse cell populations. The data suggest that chronic viral exposure and immune activation engage stress and immune signaling pathways across both epithelial and stromal lineages, potentially disrupting normal repair dynamics. Integrating the current findings with spatial transcriptomics might provide a more comprehensive picture to understand the potential effects of the altered cellular identities in HIV infection. Further work integrating longitudinal sampling, viral load quantification, and functional assay-based validations will be essential to define how these transcriptional states arise and whether they are reversible with antiretroviral therapy or targeted interventions.

## Data Availability

The datasets generated are deposited on NCBI Gene Expression Omnibus under accession code GSE319646 and is publicly available as of Mar 28, 2026. All other relevant data supporting the key findings of this study are available within the article and its supplementary files.
