# Supplementary material for: scRNA sequencing revealed HIV-associated inflammation-mediated lung epithelial dysregulation and fibroblast remodeling
Source: Front Immunol. 2026 Apr 16;17:1789140. doi: 10.3389/fimmu.2026.1789140 (PMC13128623; doi:10.3389/fimmu.2026.1789140)
Supplement: Supplementary file 1 [file SupplementaryFile1.docx]

**Supplementary Figure File**

**Title: HIV associated inflammatory remodeling of the lungs: Insights from single cell RNA sequencing**

Khursheed Ul Islam^1^, Gagandeep Kaur^1^, Sadiya Bi Shaikh^1,^ Kingshuk Panda^2^, Srinivasan Chinnapaiyan^2^, Hoshang Jehangir Unwalla^2^, and Irfan Rahman^1^

^1^Department of Environmental Medicine, University of Rochester Medical Center, Rochester, NY, USA

^2^ Department of Cellular and Molecular Medicine, Herbert Wertheim college of Medicine, Florida International university, Miami, FL 33199, USA


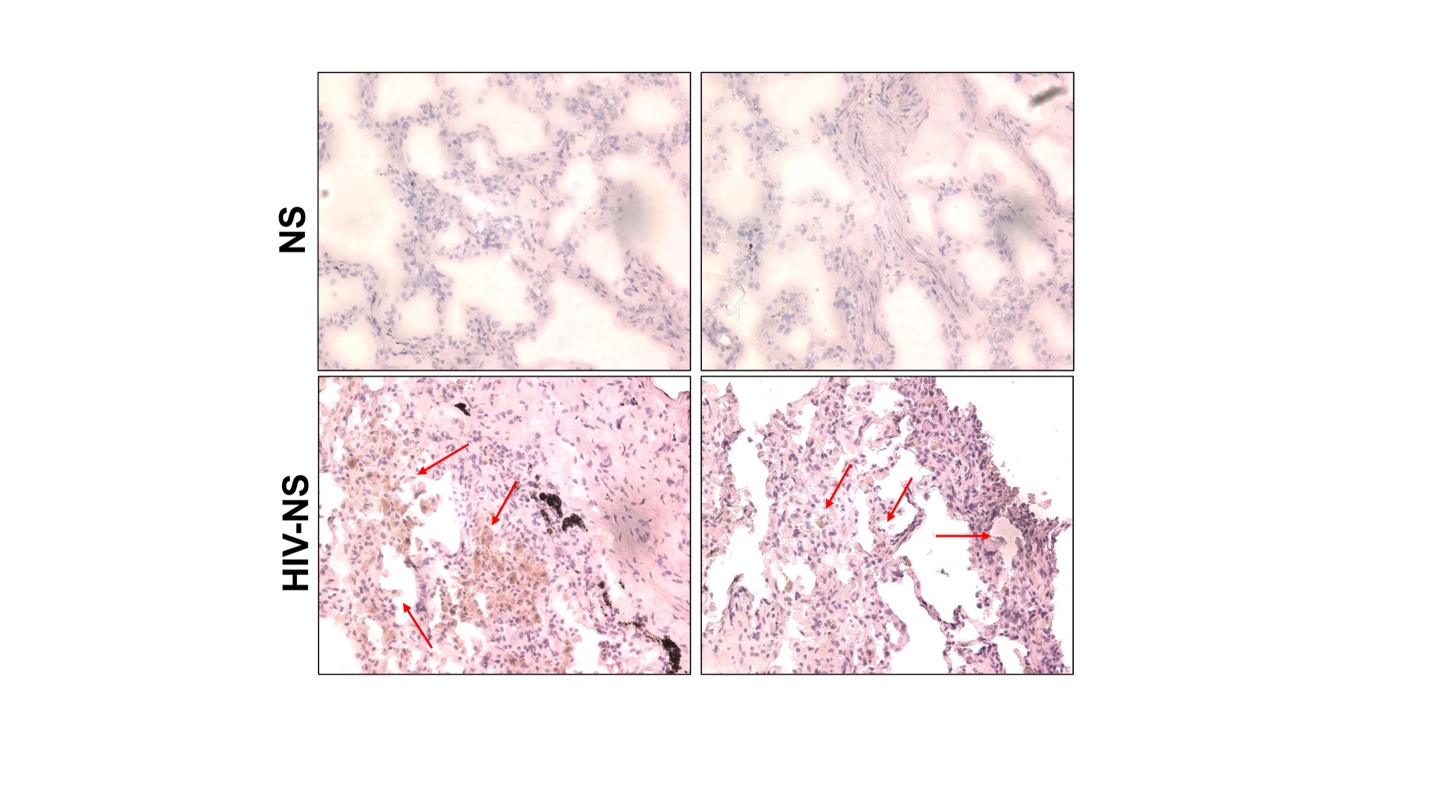


**Supplementary Figure 1**: **Detection of HIV infection in human lung tissues by IHC:** Representative images of lung sections from HIV-negative donors and HIV-positive donors stained with HIV-Tat protein using specific antibody (Catalog No. Ab42359. Abcam, USA). HIV-Positive lung sections show positive HIV-tat staining (depicted by red arrows) compared to healthy controls (HIV-negative controls). Here red arrows indicate positive Tat staining.


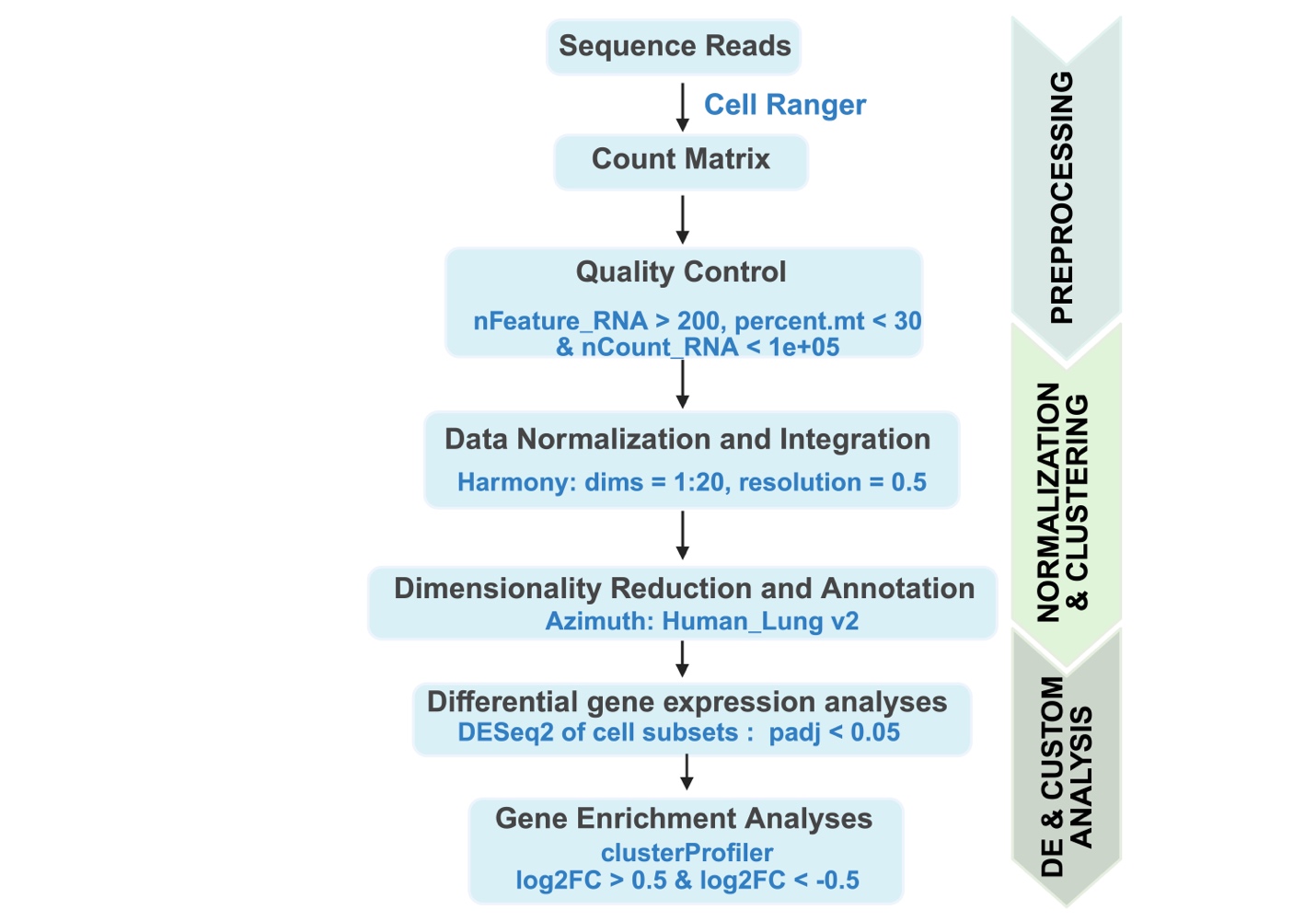


**Supplementary Figure 2**: **Flow chart depicting steps in scRNA sequencing analysis:** For analyzing the scRNA seq data Seurat v5.1.0 analyses pipeline was used. Low quality cells were excluded in quality control. Data set was integrated using RunHarmony algorithm followed by normalization of integrated data using standard Seurat pipeline. Identification of the variable genes for dimensionality reduction was done using FindvariableGenes and cell type annotation was done using Azimuth pipeline, Human_Lung v2. Finally, data set was subjected to Differential expression and custom analysis.


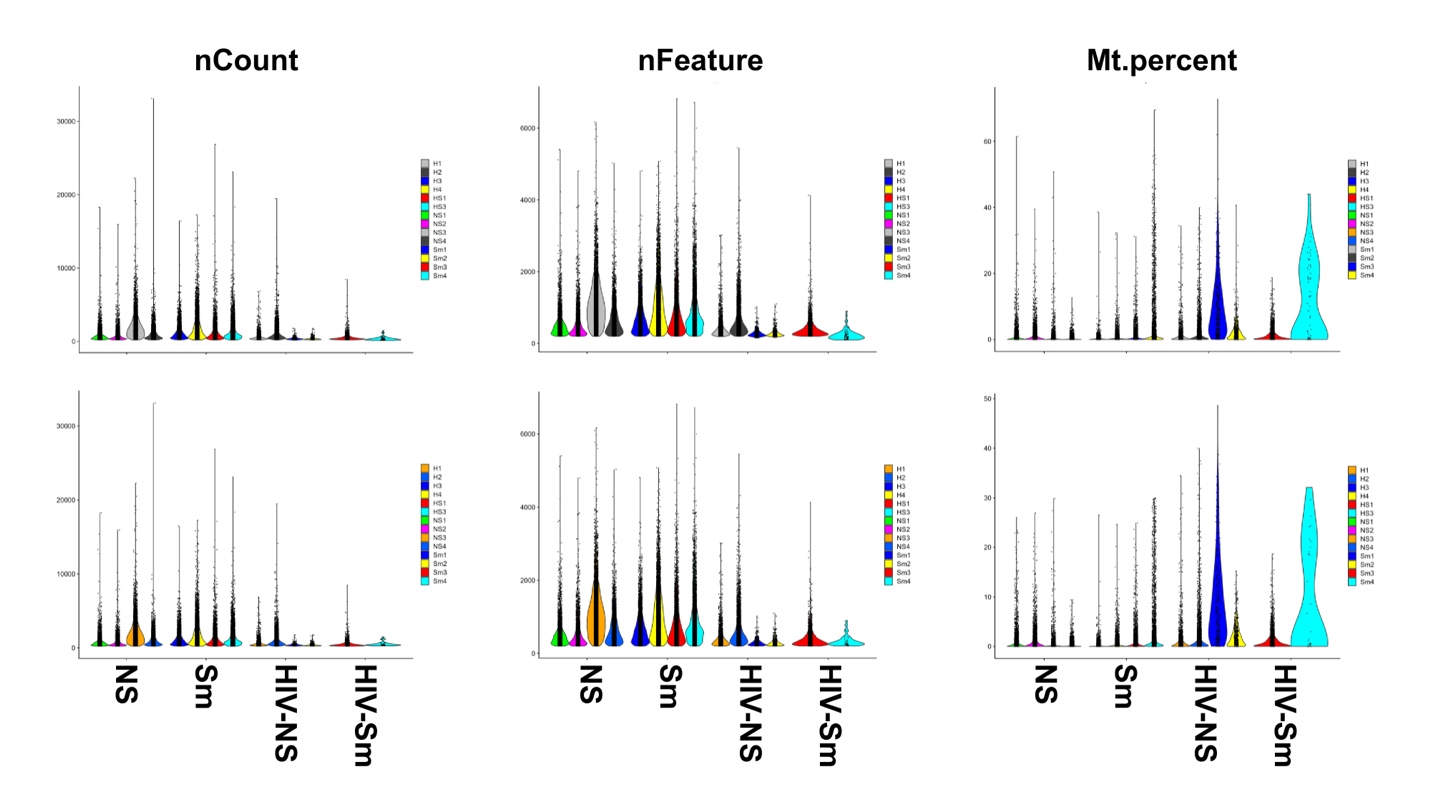


**Supplementary Figure 3: Distribution of QC metric parameters:** The violin plot depicting the distribution of QC metric parameters. A) shows the total reads/nCounts, number of features/nFeatures, and mitochondrial percentage for each sample before filtration. B) shows the total reads/nCounts, number of features/nFeatures, and mitochondrial percentage for each sample after filtration. H1, H2, H3, H4 represent non-smoker HIV lung tissues (NS-HIV samples).


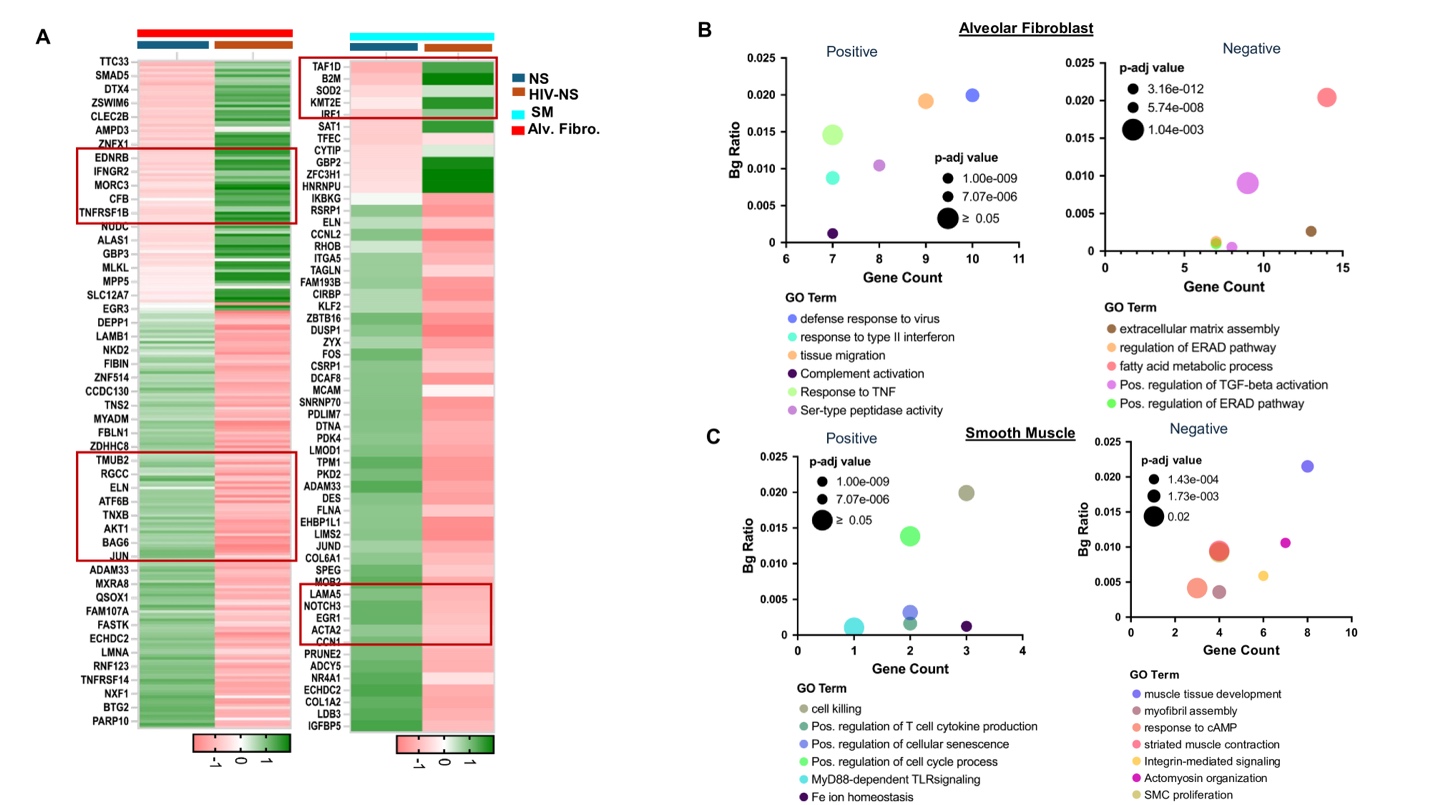


**Supplementary Figure 4:** HIV modulates stress response and extracellular matrix pathway in alveolar fibroblasts (A) Heatmap showing the significant (p<0.05) differentially enriched genes between HIV infected non-smokers and healthy controls in alveolar fibroblast and smooth muscle. (B-C) Bubble plots showing gene enrichment analyses of positively and negatively regulated genes in alveolar fibroblast and smooth muscle cell cluster from HIV-infected samples with no smoking history and healthy controls, respectively.

Full uncropped blots of E-Cadherin and β-actin corresponding to **Figure 3 (E)** in the manuscript


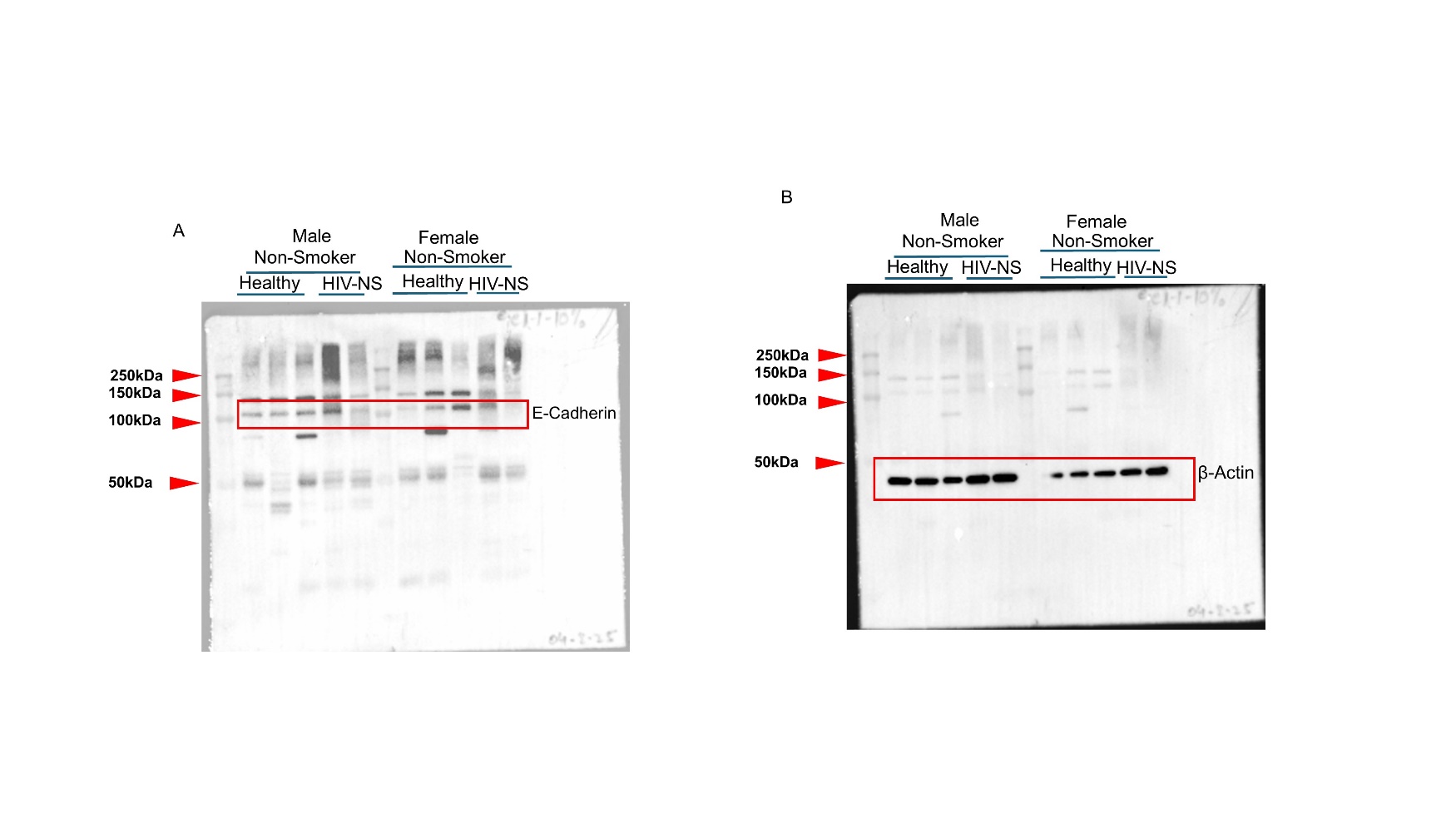


**Supplementary Figure 5**: HIV infected lung tissues show low abundance of E-cadherin protein compared to healthy controls: A) Western blot showing E-cadherin abundance in Nonsmoker healthy (NS) and Nonsmoker HIV (HIV-NS). B) Corresponding beta actin (β-actin) abundance taken as internal control.

Full uncropped blots of Fibronectin and β-actin corresponding to **Figure 3 (F)** in the manuscript


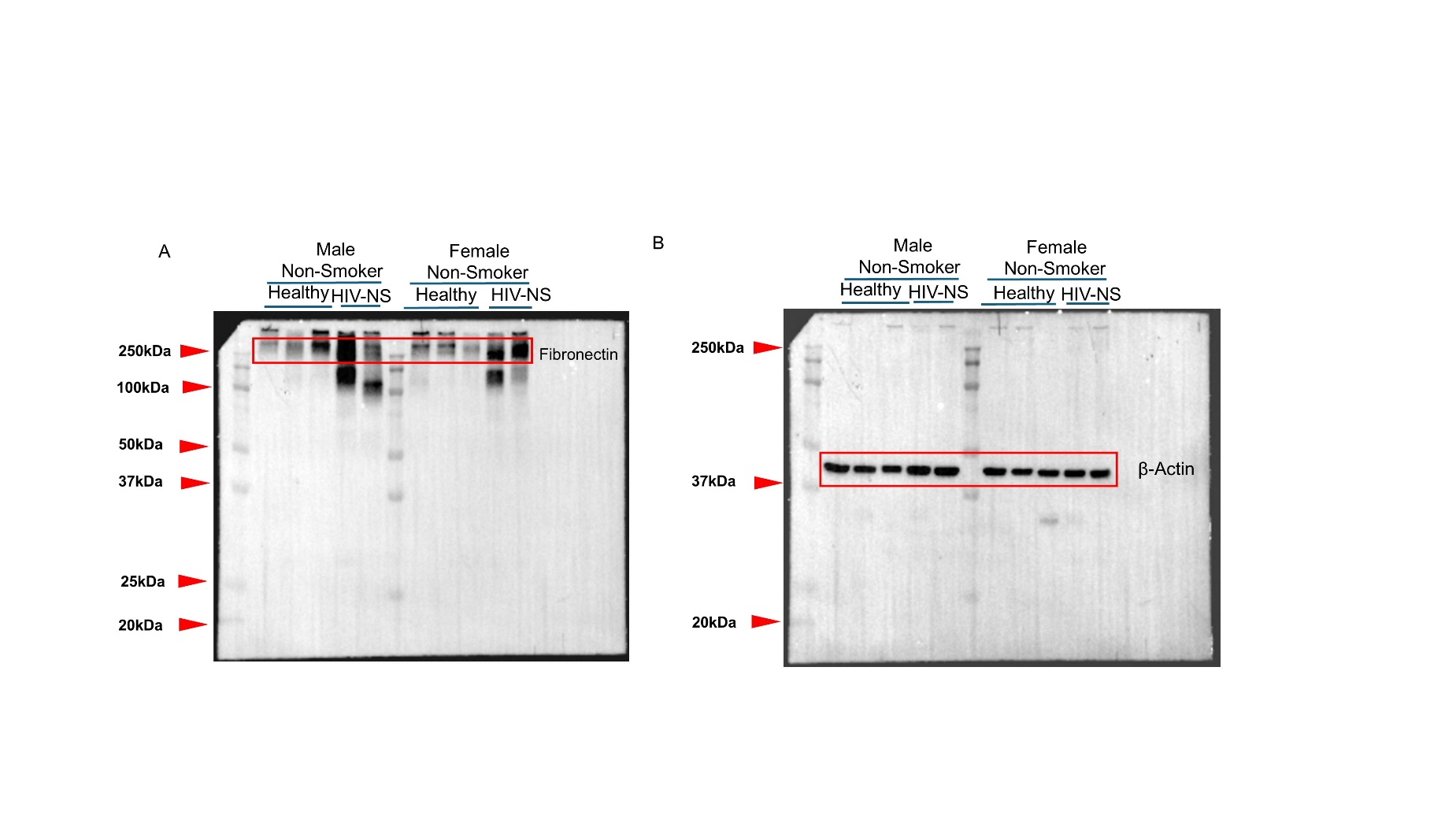


**Supplementary Figure 6**: HIV infected lung tissues show increased abundance of Fibronectin protein compared to healthy controls: A) Western blot showing Fibronectin abundance in Nonsmoker healthy (NS) and Nonsmoker HIV (HIV-NS). B) Corresponding beta actin (β-actin) abundance taken as internal control.

Full uncropped blots of Vimentin and β-actin corresponding to **Figure 3 (G)** in the manuscript


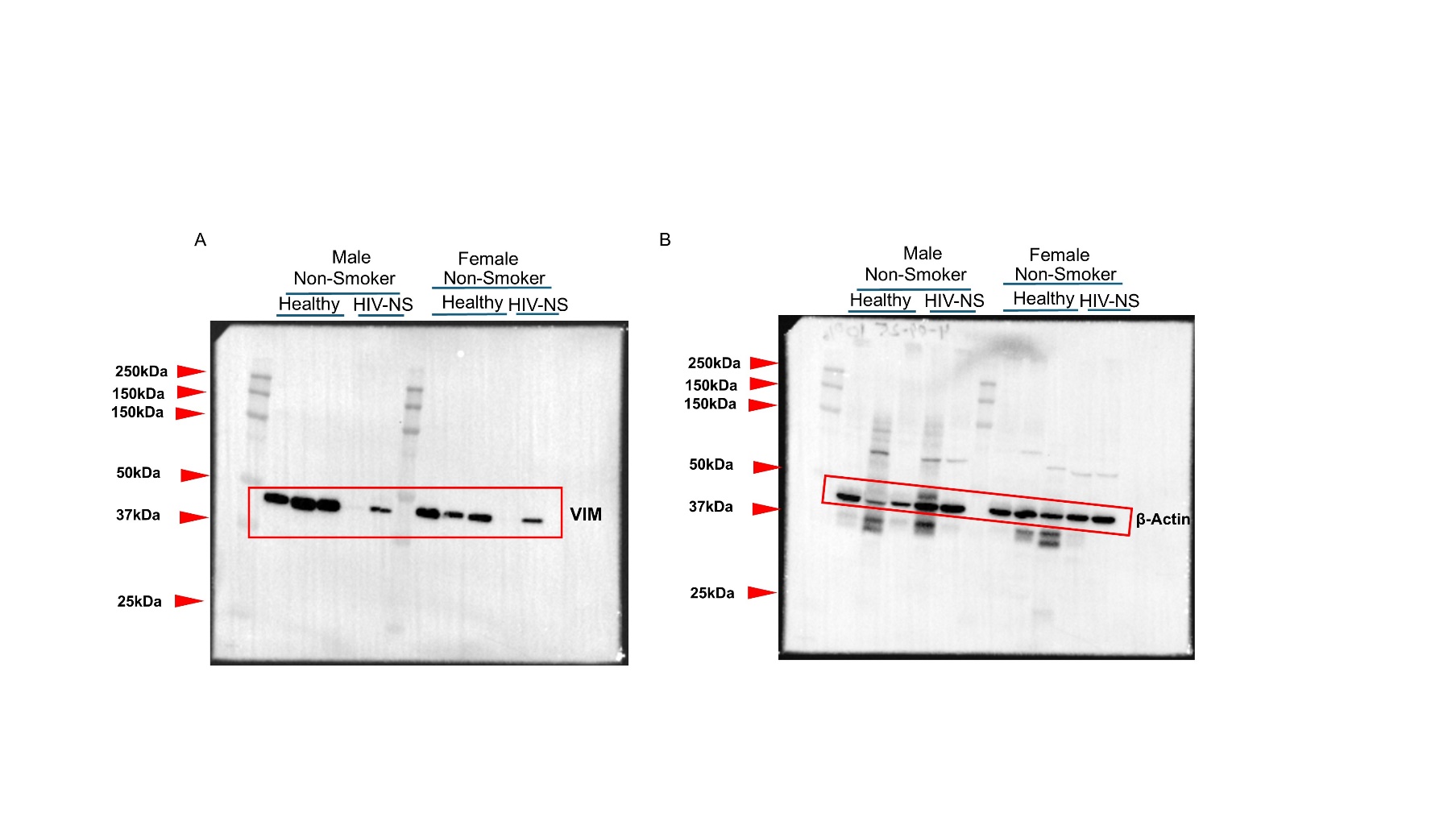


**Supplementary Figure 7**: HIV infected lung tissues show low abundance of Vimentin protein compared to healthy controls: A) Western blot showing E-cadherin abundance in Nonsmoker healthy (NS) and Nonsmoker HIV (HIV-NS). B) Corresponding beta actin (β-actin) abundance taken as internal control.
